# Cardiovascular outcomes and LDL-cholesterol levels in EMPA-REG OUTCOME^®^

**DOI:** 10.1177/1479164120975256

**Published:** 2020-12-13

**Authors:** Gisle Langslet, Bernard Zinman, Christoph Wanner, Stefan Hantel, Rosa-Maria Espadero, David Fitchett, Odd Erik Johansen

**Affiliations:** 1 Lipid Clinic, Oslo University Hospital, Oslo, Norway; 2 Lunenfeld-Tanenbaum Research Institute, Mount Sinai Hospital, Toronto, ON, Canada; 3 Division of Endocrinology, University of Toronto, Toronto, Canada; 4 Department of Medicine, Division of Nephrology, Würzburg University Clinic, Würzburg, Germany; 5 Boehringer Ingelheim Pharma GmbH & Co. KG, Biberach, Germany; 6 Boehringer Ingelheim España, S.A., Barcelona, Spain; 7 St Michael’s Hospital, Division of Cardiology, University of Toronto, Toronto, ON, Canada; 8 Boehringer Ingelheim Norway, TA Cardiometabolism, Asker, Norway; 9 Nestle Health Science SA, Cardiometabolism & Cx, Vevey, Vaud, Switzerland

**Keywords:** Type 2 diabetes mellitus, cardiovascular risk, LDL-cholesterol, SGLT-2 inhibitors, empagliflozin

## Abstract

**Objective:**

It is well established that higher low-density lipoprotein (LDL)-cholesterol levels are associated with increased cardiovascular risk. We analyzed whether effects of empagliflozin on cardiovascular outcomes varied by different LDL-cholesterol levels at baseline in EMPA-REG OUTCOME.

**Methods:**

Participants with type 2 diabetes and high cardiovascular risk received empagliflozin (10/25 mg) or placebo in addition to standard of care. We investigated the time to first 3P-MACE, cardiovascular death, hospitalization for heart failure (HHF) and all-cause mortality for empagliflozin versus placebo between baseline LDL-cholesterol categories <1.8, 1.8–<2.2, 2.2– <2.6, 2.6–3.0, and > 3.0 mmol/L, by a Cox regression including the interaction of baseline LDL-cholesterol category and treatment.

**Results:**

Of the 7020 participants randomized and treated, 81.0% received lipid lowering therapy (77.0% statins). Mean ± SD LDL-cholesterol was 2.2 ± 0.9 mmol/L, and 38%/18%, had LDL-cholesterol <1.8/>3.0 mmol/L. Age, BMI, and HbA1c levels were balanced between the LDL-cholesterol subgroups, but those in the lowest versus highest group, had more coronary artery disease (83.0% vs 59.9%) and statin treatment (88.2% vs 50.9%). Empagliflozin consistently reduced all outcomes across LDL-cholesterol categories (all interaction *p*-values > 0.05).

**Conclusion:**

The beneficial cardiovascular effects of empagliflozin was consistent across higher and lower LDL-cholesterol levels at baseline.

## Introduction

Empagliflozin is a potent and selective sodium glucose co-transporter 2 (SGLT2) inhibitor used for the treatment of type 2 diabetes (T2D). In the EMPA-REG OUTCOME trial, in patients with T2D and high CV risk, empagliflozin added to standard of care significantly reduced the risk of 3-point major adverse CV events (3P-MACE) by 14%, CV death by 38%, and hospitalization for heart failure (HHF) by 35%, compared with placebo.^1^ As there are numerous clinical and genetic studies that demonstrate a cause and effect relationship between LDL-C levels and the risk of CV events,^2,3^ the objective of this post-hoc analysis was to determine if CV outcomes with empagliflozin compared with placebo was impacted by baseline LDL-C levels in the EMPA-REG OUTCOME trial.

## Methods

### Study design and patients

The design of the EMPA-REG OUTCOME trial has been described previously.^4^ Briefly, participants with T2D (HbA1c 7.0%–9.0% (53–75 mmol/mol) for drug-naïve, 7.0%–10.0% (53–86 mmol/mol) for those on stable glucose lowering therapy), established CV disease, and estimated glomerular filtration rate (eGFR) >30 mL/min/1.73 m^2^ according to Modification of Diet in Renal Disease, were randomized to receive empagliflozin 10 mg, empagliflozin 25 mg, or placebo once daily in addition to standard of care and followed for a median of 3.1 years. Investigators were encouraged to treat CV risk factors to achieve optimal standard of care according to local guidelines. The primary outcome of the main trial was the composite outcome 3-point MACE (death from CV causes, nonfatal myocardial infarction, or nonfatal stroke), with secondary outcomes including CV death, HHF, and all-cause death, all prospectively adjudicated by Clinical Events Committees. Participants who prematurely discontinued study medication were followed for ascertainment of CV outcomes and vital status.

### Outcomes

In this post-hoc analysis, 3P-MACE, CV death, all-cause mortality and HHF were analyzed in subgroups by LDL-C levels at baseline: <1.8 mmol/L (70 mg/dL), 1.8–<2.2 mmol/L (70–<85 mg/dL), 2.2–<2.6 mmol/L (85–<100 mg/dL), 2.6–3.0 mmol/L (100–115 mg/dL), and >3.0 mmol/L (>115 mg/dL). The cut-offs for the LDL-C categories were established post-hoc on the basis to ensure a reasonable and clinical relevant range of LDL-C categories that would provide a sufficient sample size within each group to assess dose-relationships. We limited this assessment to consider only LDL-C, and not any other lipoproteins, binding protein, triglycerides, or free fatty acids.

### Statistical analysis

Analyzes compared empagliflozin pooled (10 mg and 25 mg) versus placebo in patients treated with ⩾1 dose of study drug (modified intent-to-treat population). Differences in risk between treatment groups were assessed using a Cox proportional hazards model. The model for subgroup analyzes by baseline included as factors: Sex, baseline body mass index (BMI), baseline HbA1c, baseline eGFR, region, treatment, LDL-C subgroup and treatment by baseline LDL-C interaction.

## Results

A total of 7020 participants were randomized and treated with a median observation time of 3.1 years. Baseline LDL-C information was not available for 24 participants in the placebo group and 64 in the empagliflozin group. Mean ± SD LDL-cholesterol was 2.2 ± 0.9 mmol/L, and 38%, respectively 18%, had LDL-cholesterol <1.8, or >3.0 mmol/L. LDL-C levels over time by treatment groups and LDL-C categories at baseline indicated little difference between empagliflozin and placebo (Supplemental Material Figure S1). Across the LDL-C categories there were some baseline differences (e.g. in coronary artery disease (CAD) burden, statin use, stroke, diabetes duration, blood pressure, use of antihypertensives, and proportion with albuminuria (Supplemental Material Tables S1 and S2)). Prevalence of CAD was highest in the low LDL-C subgroup (LDL-C < 1.8 mmol/L), and lowest in the high LDL-C group (LDL-C > 3.0 mmol/L), with 83.0% versus 59.9% of participants, respectively. Statin use was highest in the low LDL-C group (88.2%) and lowest in the high LDL-C (50.9%). Age, BMI, and HbA1c levels were balanced between the LDL-C subgroups.

### Lipid lowering therapy introduced post-baseline

Introduction of statin therapy was more frequent in the highest LDL-C subgroup compared with the lowest LDL-C group, 37% versus 19% of participants in the empagliflozin-group and 41.7% versus 20.2% in the placebo group, respectively, coinciding with the lower baseline prevalence of statin use in the high LDL-C subgroup. Use of other lipid lowering therapies was infrequent in all LDL-C subgroups (Supplemental Material Table S3).

### CV outcomes, HHF and all-cause mortality in subgroups by LDL-cholesterol at baseline

3-point MACE, CV mortality, and total mortality event rates varied according to LDL-C at baseline, most prominently in the placebo group (Supplemental Material Figures S2–S5). Placebo-group event rates for the primary endpoint, 3-point MACE, appeared to be highest in the two highest LDL-C subgroups (LDL-C 2.6–3.0 mmol/L and >3.0 mmol/L), with 50.8 and 54.9 events per 1000 patient-years, respectively. The lowest placebo-group 3-point MACE rate was 32.7 events per 1000 patient-years in the middle LDL-C subgroup (LDL-C 2.2–<2.6 mmol/L). In the placebo group, event rates for all outcomes, except HHF, appeared to be highest in the two highest baseline LDL-C subgroups (LDL-C >2.6 mmol/L), and lowest in the middle LDL-C subgroup.

**Figure 1.**
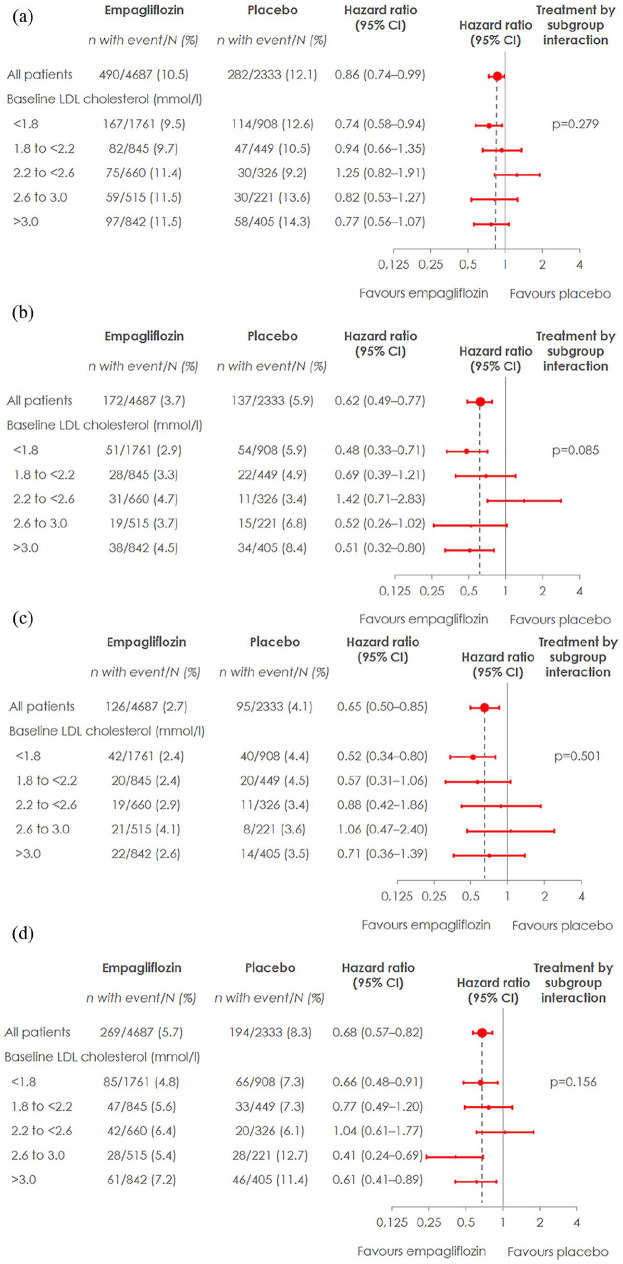
Cardiovascular outcomes, hospitalization for heart failure, and all-cause mortality by treatment group and LDL-cholesterol levels. Cox regression analysis in patients treated with ⩾1 dose of study drug: (a) 3-point MACE in subgroups by LDL-cholesterol at baseline, (b) CV death in subgroups by LDL-cholesterol at baseline, (c) hospitalization for heart failure in subgroups by LDL-cholesterol at baseline, and (d) all-cause mortality in subgroups by LDL-cholesterol at baseline.

Empagliflozin, overall, reduced all outcomes analyzed (3-point MACE, CV mortality, HHF, and total mortality), and results were consistent across categories of baseline LDL-C levels (Figure 1), as suggested by no significant interaction analyzes *p*-values (interaction *p*-values for 3P-MACE: 0.279, CV death: 0.085, HHF: 0.501, and all-cause mortality: 0.156).

### Adverse events

Adverse events during trial follow-up across LDL-categories at baseline are reported in Supplemental Material Table S4, and were consistent with observations reported previously.^1^

## Discussion

Our analysis shows that the modulating beneficial effects of empagliflozin on CV outcomes did not differ between the categories of baseline LDL-C levels.

This consistency of effects of empagliflozin is reassuring for several reasons. First, consistent with overall effects, protective effects is likely also be expected in those with low LDL-C levels, which many patients now accomplish with use of high-dose statins, combined, if needed, with ezetimibe and/or inhibitors of proprotein convertase subtilisin/kexin type 9 (PCSK9),^5^ although the latter was not studied in EMPA-REG OUTCOME. Second, it is well established the SGLT-2 inhibitors contribute to a modest or small increase in LDL-C levels, as well as other lipoproteins, primarily due to hemoconcentration,^6^ although alternative hypotheses are also proposed like decreased LDL receptors expression on the surface of hepatocytes,^7^ however this increase does not offset their treatment effect.

An interesting observation was that there appeared to be a J-shaped relation between baseline LDL-C levels and rates of 3-point MACE, CV mortality and total mortality in the placebo group, and both J-shaped relationships,^8^ and linear relationships,^9,3^ between LDL-C or total cholesterol and mortality have been reported by others. According to the inclusion criteria, in addition to T2D, all patients enrolled in the study should have established CV disease. Those in the lowest baseline LDL-C group (LDL-C < 1.8 mmol/L) had higher baseline prevalence of CAD and statin use than those in the higher LDL-C groups, reflecting more advanced CV disease that are treated more aggressively with lipid lowering treatment, as per guideline recommendations. Based on their high baseline CV risk, one would expect the subjects within this group to have the highest CV event rates, however this was observed in the placebo-group with the highest LDL-C levels, even though they had less CAD prevalence. Since the trial did not perform a stratified randomization according to LDL-C levels, we can only speculate if this a confounder effect related to the lower statin use at baseline, as only 66% of those with LDL-C < 2.6–3.0 mmol/L, and 51% of those with LDL-C > 3.0 mmol/L received statins, which is low in a patient population with T2D and CV disease. These results therefore also underscore the necessity to more actively consider statins as part of the preventive treatment regimen in T2D, as randomized, secondary prevention, placebo-controlled studies, consistently have demonstrated that statin use reduce the risk of recurrence of CV disease.^10^

A strength of this post-hoc analysis is the relatively large number of participants, yielding a relatively high number of patients in each of the LDL-C subgroups. The post-hoc nature of the analysis however, limit the validity of the results. Further, PCSK9-ihibitors were not available at the time of trial conduct, and we did not capture details around doses of lipid-lowering therapies introduced, for example, doses of various statins, or doses of omega-3 fatty acids, however, given the consistency of effects, we do not think that this influences the interpretation.

In conclusion, the beneficial cardiovascular effects of empagliflozin did not differ between the categories of baseline LDL-C. A relatively high CV event rate and a low prevalence of statin use among participants within the highest baseline LDL-C subgroups also underscores the importance of aggressive lipid-lowering therapy in high CV risk populations.

## Supplemental Material

sj-pdf-1-dvr-10.1177_1479164120975256 – Supplemental material for Cardiovascular outcomes and LDL-cholesterol levels in EMPA-REG OUTCOME^®^ Click here for additional data file. Supplemental material, sj-pdf-1-dvr-10.1177_1479164120975256 for Cardiovascular outcomes and LDL-cholesterol levels in EMPA-REG OUTCOME^®^ by Gisle Langslet, Bernard Zinman, Christoph Wanner, Stefan Hantel, Rosa-Maria Espadero, David Fitchett and Odd Erik Johansen in Diabetes & Vascular Disease Research
