# Supplementary material for: Cardiovascular outcomes and LDL-cholesterol levels in EMPA-REG OUTCOME®
Source: Diab Vasc Dis Res. 2020 Dec 13;17(6):1479164120975256. doi: 10.1177/1479164120975256 (PMC7919220; doi:10.1177/1479164120975256)
Supplement: sj-pdf-1-dvr-10.1177_1479164120975256 – Supplemental material for Cardiovascular outcomes and LDL-cholesterol levels in EMPA-REG OUTCOME® [file sj-pdf-1-dvr-10.1177_1479164120975256.pdf]

Supplementary Online Content

Gisle Langslet, Bernard Zinman, Christoph Wanner, Stefan Hantel, Rosa-Maria Espadero, David Fitchett, Odd Erik Johansen. Cardiovascular Outcomes According to LDL-Cholesterol Levels in EMPA-REG OUTCOME®

	Content	Page
Figure S1	LDL cholesterol over time by treatment group and LDL cholesterol levels at baseline	2-3
Table S1	Baseline characteristics in subgroups by LDL cholesterol at baseline	4
Table S2	Baseline CV risk factors and CV medications in subgroups by LDL cholesterol at baseline	5
Table S3	Lipid lowering therapy introduced post-baseline	6
Figure S2-S5	Incidence rates for 3P-MACE, CV death, hospitalized heart failure and all-cause mortality by treatment with placebo or empagliflozin by LDL-cholesterol categories at baseline	7-8
Table S4	Adverse events occurring until 7 days after treatment discontinuation in participants in EMPA-REG OUTCOME by treatment group and by LDL-cholesterol levels at baseline.	9

Figure S1. LDL cholesterol over time by empagliflozin and placebo treatment groups, and LDL cholesterol at baseline. Data are adjusted mean ± standard error in the treated set.

The model includes baseline HbA1c as linear covariate(s) and baseline eGFR category, baseline BMI category, geographical region, week reachable lipid parameter, treatment, visit, baseline LDL category (mg/dL), treatment by visit interaction, visit by baseline LDL category (mg/dL) interaction, treatment by baseline LDL category (mg/dL) interaction, treatment by visit by baseline LDL category (mg/dL) interaction, baseline HbA1c by visit interaction as fixed effect(s).

A. LDL-C <1.8 mmol/L [70 mg/dL] at baseline

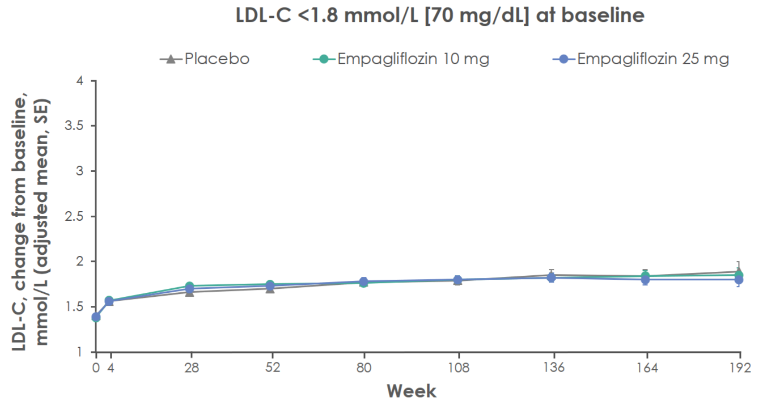

Placebo	872	866	587	427	319	229	147	91	42
Empagliflozin 10 mg	842	835	645	516	416	331	222	150	91
Empagliflozin 25 mg	838	830	632	492	406	339	219	152	81

B. LDL-C 1.8 – <2.2 mmol/L [70-<85 mg/dL] at baseline

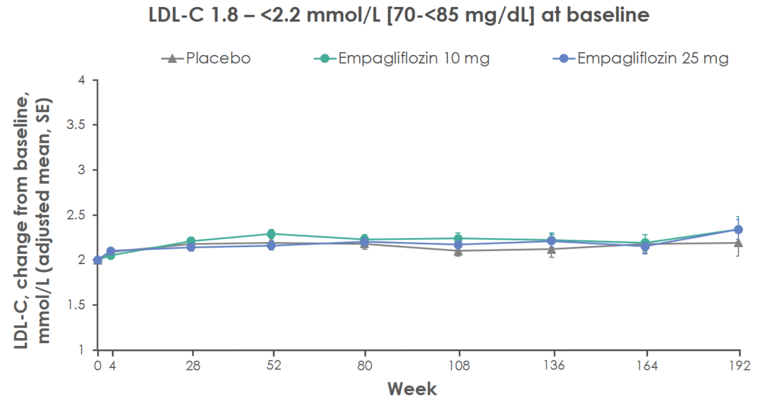

Placebo	432	429	294	218	170	122	67	43	25
Empagliflozin 10 mg	394	389	304	242	194	153	85	57	28
Empagliflozin 25 mg	423	418	312	252	209	163	111	80	44

Empagliflozin efficacy by baseline LDL-C levels

C. LDL-C 2.2 – <2.6 mmol/L [85-<100 mg/dL] at baseline

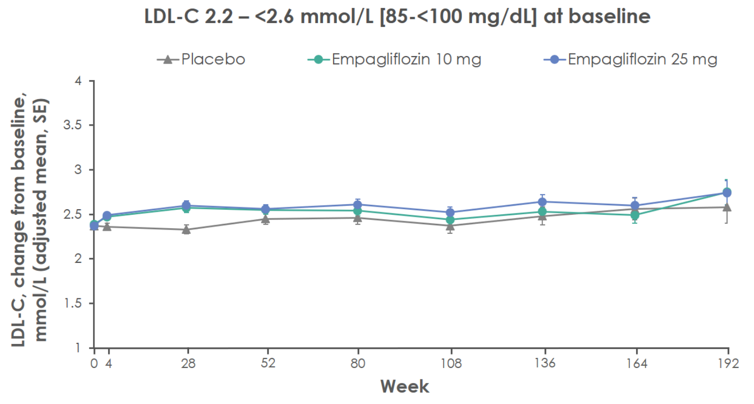

	0	4	28	52	80	108	136	164	192
Placebo	310	308	204	137	95	76	53	32	17
Empagliflozin 10 mg	324	320	247	203	164	132	83	60	28
Empagliflozin 25 mg	313	309	227	182	146	129	89	65	30

D. LDL-C 2.6 – <3.0 mmol/L [100-<115 mg/dL] at baseline

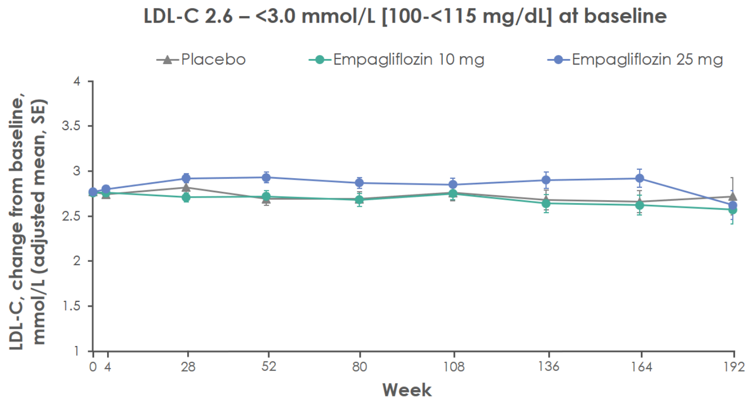

	0	4	28	52	80	108	136	164	192
Placebo	212	210	155	115	85	64	41	32	12
Empagliflozin 10 mg	248	245	193	151	122	103	58	45	22
Empagliflozin 25 mg	243	240	195	156	133	107	69	55	23

E. LDL-C > 3.0 mmol/L [>115 mg/dL] at baseline

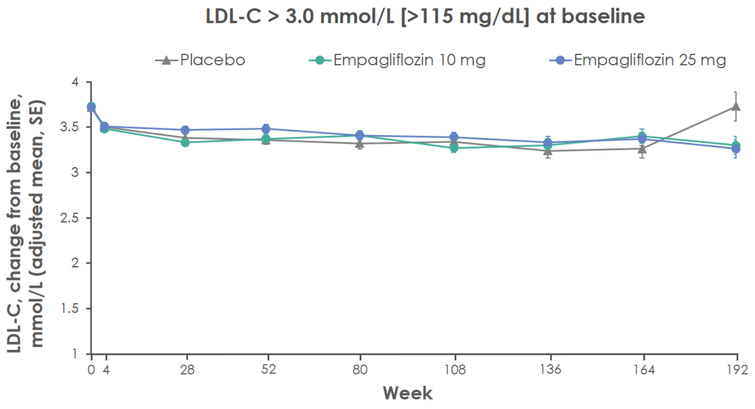

	0	4	28	52	80	108	136	164	192
Placebo	393	390	269	201	153	129	73	52	23
Empagliflozin 10 mg	413	410	312	263	213	189	117	85	57
Empagliflozin 25 mg	394	391	312	257	218	189	117	96	56

Table S1. Baseline characteristics in subgroups by LDL cholesterol at baseline. Data are n (%) or mean ± SD in patients treated with ≥1 dose of study drug.

	**All patients (N=7020)**	**LDL cholesterol at baseline***				
		**<1.8 mmol/L**	**1.8 to <2.2 mmol/L**	**2.2 to <2.6 mmol/L**	**2.6 to 3.0 mmol/L**	**>3.0 mmol/L**
		<70 mg/dL (N=2669)	70 to <85 mg/dL (N=1294)	85 to <100 mg/dL (N=986)	100 to 115 mg/dL (N=736)	>115 mg/dL (N=1247)
Male	5016 (71.5)	2082 (78.0)	957 (74.0)	709 (71.9)	486 (66.0)	723 (58.0)
Age, years	63.1 ± 8.6	63.7 ± 8.6	63.8 ± 8.4	62.6 ± 8.5	62.6 ± 8.8	62.0 ± 8.8
Body Mass Index, kg/m^2	30.6 ± 5.3	30.7 ± 5.2	30.7 ± 5.2	30.7 ± 5.4	30.6 ± 5.2	30.2 ± 5.2
LDL cholesterol, mmol/L	2.22 ± 0.92	1.39 ± 0.31	2.00 ± 0.10	2.38 ± 0.11	2.77 ± 0.11	3.74 ± 0.72
LDL cholesterol, mg/dL	85.6 ± 35.7	53.8 ± 11.9	77.4 ± 4.0	91.9 ± 4.4	107.0 ± 4.2	144.3±27.8
SBP <140 mmHg and DBP <90 mmHg	4306 (61.3)	1715 (64.3)	811 (62.7)	581 (58.9)	436 (59.2)	710 (56.9)
eGFR (MDRD), $mL/min/1.73m^2$	74.0 ± 21.4	73.3 ± 21.0	74.4 ± 21.2	74.0 ± 20.9	74.7 ± 22.2	74.9 ± 22.6
UACR (mg/g)						
Normal (< 30)	4171 (59.4)	1645 (61.6)	791 (61.1)	584 (59.2)	437 (59.4)	661 (53.0)
Microalbum. (30-300)	2013 (28.7)	751 (28.1)	357 (27.6)	286 (29.0)	212 (28.8)	382 (30.6)
Macroalbum. (> 300)	769 (11.0)	251 (9.4)	131 (10.1)	109 (11.1)	81 (11.0)	193 (15.5)
HbA1c**, %	8.07 ± 0.85	8.02 ± 0.82	8.04 ± 0.83	8.09 ± 0.87	8.11 ± 0.88	8.17 ± 0.89
Time since diagnosis of type 2 diabetes, years						
≤1	180 (2.6)	60 (2.2)	28 (2.2)	28 (2.8)	16 (2.2)	45 (3.6)
>1 to 5	1083 (15.4)	340 (12.7)	181 (14.0)	165 (16.7)	138 (18.8)	243 (19.5)
>5 to 10	1746 (24.9)	664 (24.9)	306 (23.6)	229 (23.2)	186 (25.3)	340 (27.3)
>10	4011 (57.1)	1605 (60.1)	779 (60.2)	564 (57.2)	396 (53.8)	619 (49.6)

*Baseline LDL cholesterol information was not available for 88 patients. ** Data were not available for 2 patients. Abbreviations: DBP, diastolic blood pressure; SBP, systolic blood pressure; eGFR, estimated glomerular filtration rate; MDRD, Modification of Diet in Renal Disease formula, UACR, Urine albumin-to-creatinine ratio, album; albuminuria.

Table S2. Baseline CV risk factors and CV medications in subgroups by LDL cholesterol at baseline. Data are n (%) or mean ± SD in patients treated with ≥1 dose of study drug.

	**All patients (N=7020)**	**LDL cholesterol at baseline***				
		**<1.8 mmol/L**	**1.8 to <2.2 mmol/L**	**2.2 to <2.6 mmol/L**	**2.6 to 3.0 mmol/L**	**>3.0 mmol/L**
		**<70 mg/dL (N=2669)**	**70 to <85 mg/dL (N=1294)**	**85 to <100 mg/dL (N=986)**	**100 to 115 mg/dL (N=736)**	**>115 mg/dL (N=1247)**
Any CV risk factor	6964 (99.2)	2652 (99.4)	1283 (99.1)	977 (99.1)	731 (99.3)	1235 (99.0)
CAD	5308 (75.6)	2215 (83.0)	1035 (80.0)	745 (75.6)	502 (68.2)	747 (59.9)
Multi-vessel CAD	3279 (46.7)	1410 (52.8)	665 (51.4)	467 (47.4)	289 (39.3)	409 (32.8)
History of MI	3273 (46.6)	1351 (50.6)	607 (46.9)	454 (46.0)	314 (42.7)	514 (41.2)
Coronary artery bypass graft	1738 (24.8)	736 (27.6)	362 (28.0)	228 (23.1)	159 (21.6)	231 (18.5)
History of stroke**	1637 (23.3)	531 (19.9)	248 (19.2)	213 (21.6)	215 (29.2)	409 (32.8)
Peripheral artery disease	1461 (20.8)	511 (19.1)	248 (19.2)	212 (21.5)	149 (20.2)	316 (25.3)
Single vessel CAD*	736 (10.5)	326 (12.2)	147 (11.4)	111 (11.3)	64 (8.7)	81 (6.5)
Cardiac failure†	706 (10.1)	250 (9.4)	119 (9.2)	90 (9.1)	84 (11.4)	154 (12.3)
Anti-hypertensive therapy	6667 (95.0)	2582 (96.7)	1245 (96.2)	934 (94.7)	691 (93.9)	1132 (90.8)
ACE inhibitors/ARBs	5666 (80.7)	2208 (82.7)	1071 (82.8)	792 (80.3)	566 (76.9)	954 (76.5)
Statins	5403 (77.0)	2353 (88.2)	1090 (84.2)	768 (77.9)	488 (66.3)	635 (50.9)
Acetylsalicylic acid	5803 (82.7)	2306 (86.4)	1111 (85.9)	826 (83.8)	572 (77.7)	915 (73.4)

*Baseline LDL cholesterol information was not available for 88 patients. ** Information was not available for 1 patient. †Based on the narrow standard MedDRA query 'cardiac failure'. Abbreviations: ACE, angiotensin-converting enzyme; ARB, angiotensin receptor blocker; CAD, coronary artery disease; MI, myocardial infarction.

Table S3. Lipid-lowering therapy introduced post-baseline. Data are n (%) in patients treated with ≥1 dose of study drug.

	**LDL cholesterol at baseline***									
	**<1.8 mmol/L**		**1.8 to <2.2 mmol/L**		**2.2 to <2.6 mmol/L**		**2.6 to 3.0 mmol/L**		**>3.0 mmol/L**	
	**<70 mg/dL**		**70 to <85 mg/dL**		**85 to <100 mg/dL**		**100 to 115 mg/dL**		**>115 mg/dL**	
	Placebo (N=908)	Empa (N=1761)	Placebo (N=449)	Empa (N=845)	Placebo (N=326)	Empa (N=660))	Placebo (N=221)	Empa (N=515)	Placebo (N=405)	Empa (N=842)
Lipid-lowering therapy	223 (24.6)	424 (24.1)	121 (26.9)	224 (26.5)	107 (32.8)	201 (30.5)	78 (35.3)	158 (30.7)	184 (45.4)	338 (40.1)
Statins	183 (20.2)	334 (19.0)	98 (21.8)	183 (21.7)	83 (25.5)	169 (25.6)	63 (28.5)	129 (25.0)	169 (41.7)	311 (36.9)
Fibrates	36 (4.0)	83 (4.7)	20 (4.5)	37 (4.4)	29 (8.9)	34 (5.2)	11 (5.0)	21 (4.1)	32 (7.9)	33 (3.9)
Ezetimibe	12 (1.3)	24 (1.4)	8 (1.8)	21 (2.5)	14 (4.3)	13 (2.0)	5 (2.3)	13 (2.5)	9 (2.2)	21 (2.5)
Niacin	6 (0.7)	6 (0.3)	4 (0.9)	5 (0.6)	1 (0.3)	2 (0.3)	1 (0.5)	4 (0.8)	3 (0.7)	3 (0.4)
Other**	29 (3.2)	41 (2.3)	11 (2.4)	26 (3.1)	8 (2.5)	8 (1.2)	10 (4.5)	15 (2.9)	13 (3.2)	18 (2.1)

*Baseline LDL cholesterol information was not available for 24 patients in the placebo group and 64 patients in the empagliflozin group.**Includes omega-3 fatty acids and bile acid sequestrants.

Empagliflozin efficacy by baseline LDL-C levels

Figure S2. 3-point MACE event rates by LDL-cholesterol levels at baseline in EMPA-REG OUTCOME.

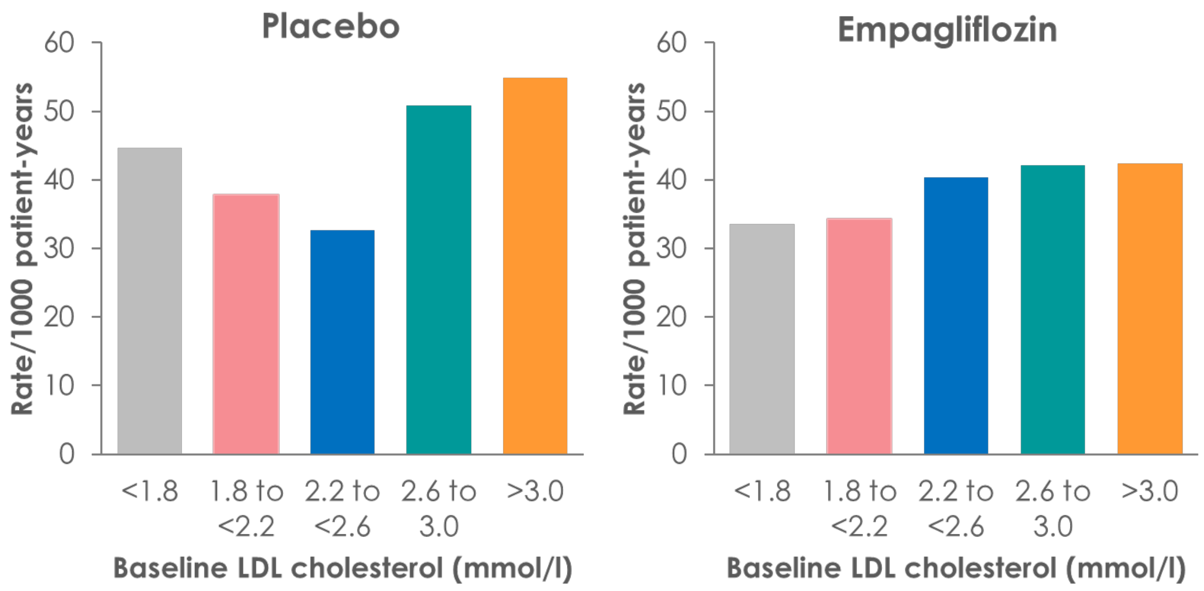

Figure S3. CV death event rates by LDL-cholesterol levels at baseline in EMPA-REG OUTCOME

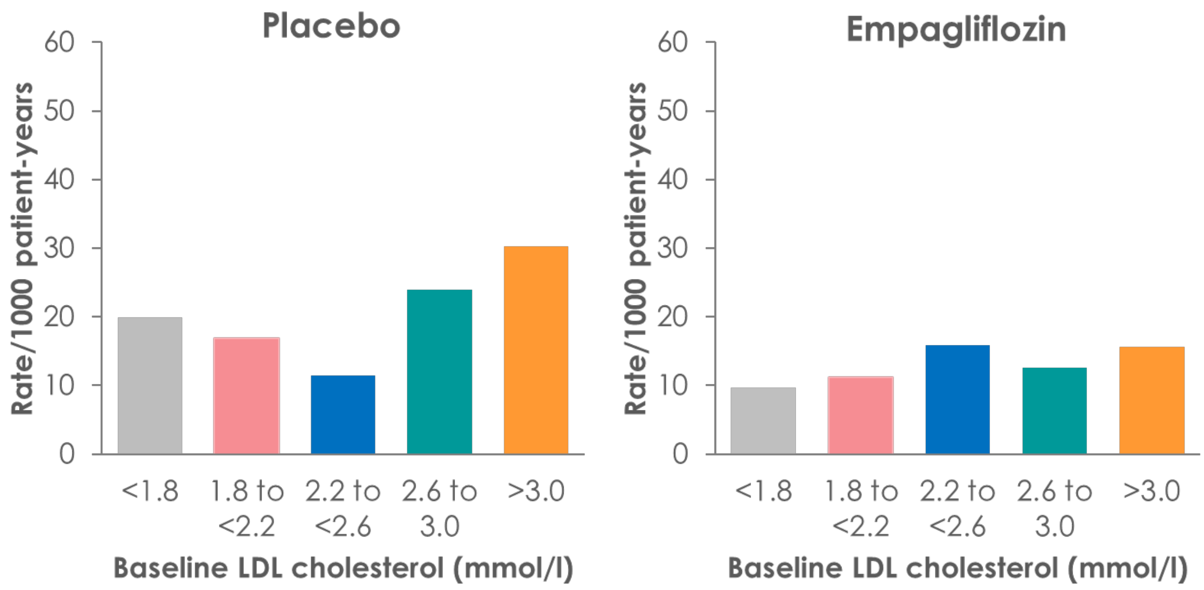

Figure S4. Hospitalization for heart failure event rates by LDL-cholesterol levels at baseline in EMPA-REG OUTCOME

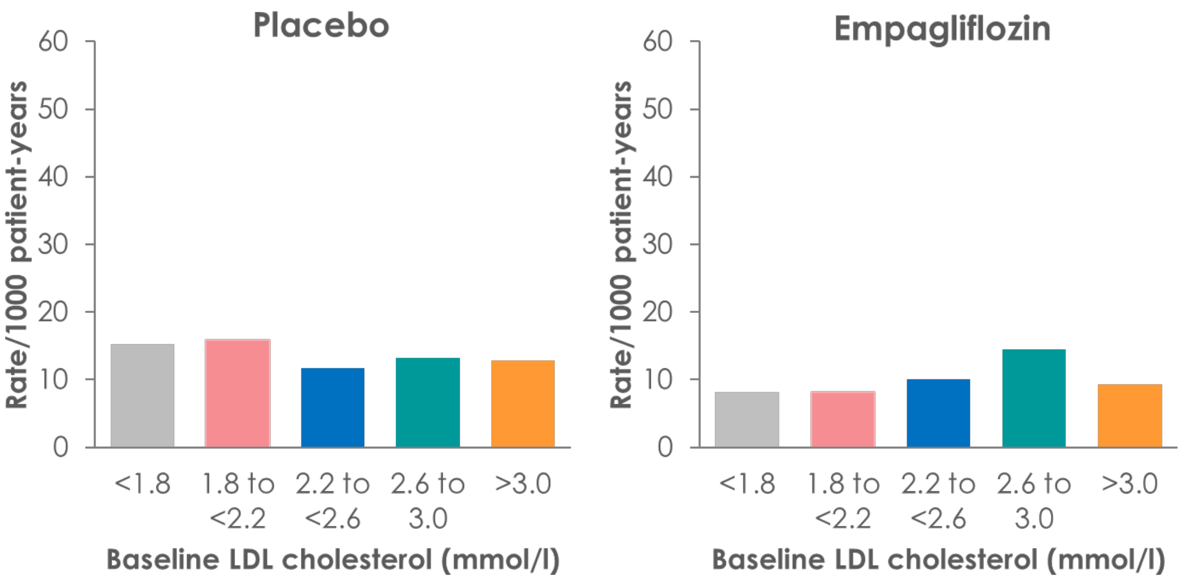

Figure S5. All-cause mortality event rates by LDL-cholesterol levels at baseline in EMPA-REG OUTCOME

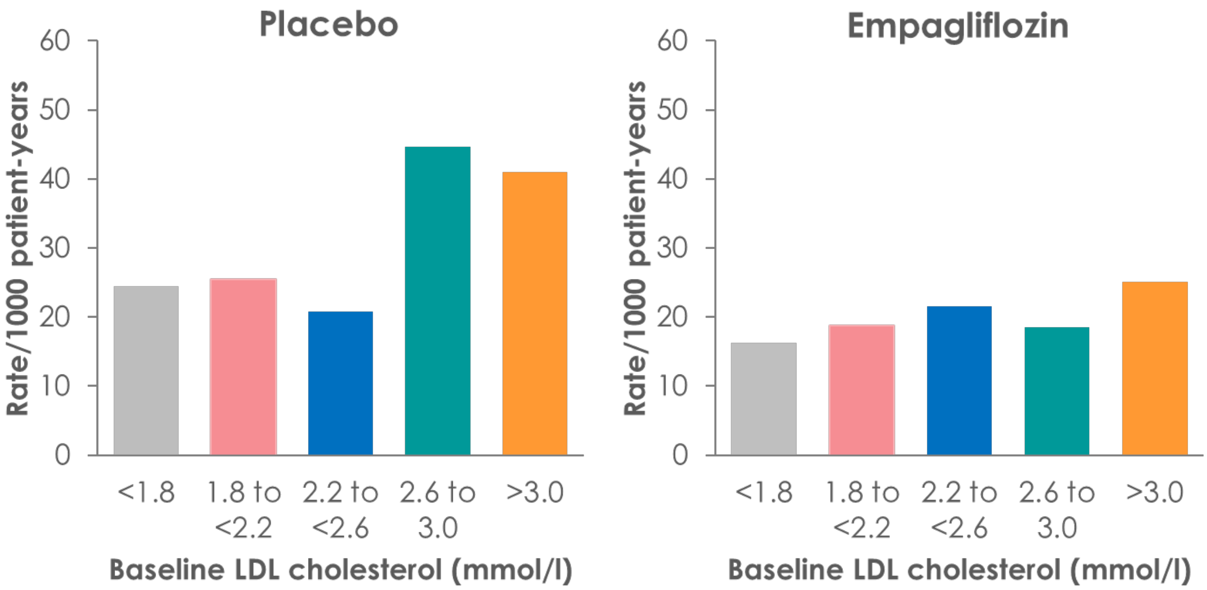

Table S4 Adverse events occurring until 7 days after treatment discontinuation in participants in EMPA-REG OUTCOME by treatment group and by LDL-cholesterol levels at baseline.

	**LDL cholesterol at baseline***									
	**<1.8 mmol/L**		**1.8 to <2.2 mmol/L**		**2.2 to <2.6 mmol/L**		**2.6 to 3.0 mmol/L**		**>3.0 mmol/L**	
	**<70 mg/dL**		**70 to <85 mg/dL**		**85 to <100 mg/dL**		**100 to 115 mg/dL**		**>115 mg/dL**	
	**Placebo (N=908)**	**Empa (N=1761)**	**Placebo (N=449)**	**Empa (N=845)**	**Placebo (N=326)**	**Empa (N=660)**	**Placebo (N=221)**	**Empa (N=515)**	**Placebo (N=405)**	**Empa (N=842)**
One or more AEs	838 (92.3)	1612 (91.5)	411 (91.5)	779 (92.2)	306 (93.9)	586 (88.8)	192 (86.9)	458 (88.9)	371 (91.6)	733 (87.1)
One or more serious AEs	400 (44.1)	686 (39.0)	189 (42.1)	332 (39.3)	130 (39.9)	257 (38.9)	97 (43.9)	193 (37.5)	164 (40.5)	295 (35.0)
One or more AEs leading to discontinuation of trial drug	176 (19.4)	305 (17.3)	74 (16.5)	157 (18.6)	62 (19.0)	121 (18.3)	41 (18.6)	83 (16.1)	94 (23.2)	138 (16.4)
Confirmed hypoglycaemic AEs**	292 (32.2)	509 (28.9)	107 (23.8)	272 (32.2)	103 (31.6)	183 (27.7)	52 (23.5)	135 (26.2)	90 (22.2)	183 (21.7)
Hypoglycaemia requiring assistance	19 (2.1)	21 (1.2)	5 (1.1)	13 (1.5)	7 (2.1)	10 (1.5)	3 (1.4)	10 (1.9)	2 (0.5)	8 (1.0)
Event consistent with UTI***	148 (16.3)	294 (16.7)	62 (13.8)	137 (16.2)	62 (19.0)	121 (18.3)	45 (20.4)	102 (19.8)	98 (24.2)	176 (20.9)
Event consistent with genital infection†	20 (2.2)	114 (6.5)	7 (1.6)	64 (7.6)	2 (0.6)	46 (7.0)	6 (2.7)	31 (6.0)	6 (1.5)	43 (5.1)
Event consistent with volume depletion‡	55 (6.1)	102 (5.8)	27 (6.0)	41 (4.9)	15 (4.6)	33 (5.0)	12 (5.4)	26 (5.0)	5 (1.2)	35 (4.2)
Acute renal failure§	69 (7.6)	95 (5.4)	28 (6.2)	48 (5.7)	21 (6.4)	33 (5.0)	11 (5.0)	26 (5.0)	26 (6.4)	43 (5.1)
Thromboembolic event¶	4 (0.4)	12 (0.7)	5 (1.1)	4 (0.5)	4 (1.2)	4 (0.6)	1 (0.5)	4 (0.8)	5 (1.2)	6 (0.7)
Bone fracture¦	31 (3.4)	69 (3.9)	20 (4.5)	35 (4.1)	13 (4.0)	20 (3.0)	8 (3.6)	19 (3.7)	18 (4.4)	31 (3.7)

n (%) of patients treated with ≥1 dose of study drug, based on events that occurred during treatment or ≤7 days after last intake of study drug. *Baseline LDL cholesterol information was not available for 24 patients in the placebo group and 64 patients in the empagliflozin group. **Plasma glucose ≤ 3.9 mmol/l and/or requiring assistance. ***Based on 79 MedDRA preferred terms. †Based on 88 MedDRA preferred terms. ‡Based on 8 MedDRA preferred terms. §Based on narrow standardised MedDRA query “acute renal failure”. ¶Based on narrow standardised MedDRA query “embolic and thrombotic events”. ¦Based on 62 MedDRA preferred terms. MedDRA, Medical Dictionary for Regulatory Activities. Empa – Empagliflozin. AE – adverse events.
